# Linguistic characteristics in bipolar disorder versus borderline personality disorder

**DOI:** 10.1038/s41598-023-46038-7

**Published:** 2023-12-07

**Authors:** Noelia Santos Muriel, Patricia López Resa, Esther Moraleda Sepúlveda

**Affiliations:** 1 Department of Psychology, Faculty of Health Sciences, University of Castilla-La-Mancha, Avda Real Fábrica de la Seda s/n, 45600 Talavera de la Reina, Spain; 2 Department of Psychology, Faculty of Pychology, University Complutense, Campus de Somosaguas, 28223 Pozuelo de Alarcón, Madrid, Spain

**Keywords:** Health care, Medical research

## Abstract

Scientific evidence has documented throughout the research carried out in recent years, the neuropsychological, behavioral and adaptive difficulties presented by people with Bipolar Disorder and Borderline Personality Disorder at different stages of their development. However, little importance has been given to other factors such as communication, especially in the adult population. The objective of this research was to know the language characteristics presented by people from both groups and the differences in linguistic development. The sample consisted of 60 participants between the ages of 17 and 42:31 of them with a diagnosis of Borderline Personality Disorder and the remaining 29 with a diagnosis of Bipolar Disorder. The standardized evaluation instruments were: the Social Skills Scale and the Pragmatic Competence Questionnaire completed by three different informants (families, professionals and the own person). The results obtained show that both populations manifest linguistic difficulties in adulthood and that there are differences depending on the perception of the agent involved in the language assessment. These results are highly relevant since they provide up-to-date information about language level, support the need for language intervention in adulthood, and reflect a different communicative profile in Bipolar Disorder and Borderline Personality Disorder.

## Introduction

Thinking is the function through which cognitions are created and fostered in human beings from infancy, which is based on the experiences that have shaped their lives, their environment, and the people around them^1^. Therefore, a distortion in thinking, such as in the case of mental disorders, will lead to a distortion in language^2^, resulting in what we know as dysfunctional thoughts or cognitive distortions^3,4^. Taking these characteristics into account, it can be affirmed that Bipolar Disorder (BD hereafter) and Borderline Personality Disorder (BPD hereafter) can be encompassed within this category. Various research studies have shown that the language deficits present in mental disorders are due to distortions in thinking and cognitive impairments, as is characteristic in both BD and schizophrenia^5^. Furthermore, it appears that there is an increase in cognitive distortions in the population through linguistic evaluation^6^. In this regard, some studies suggest the importance and significant impact of considering language as a biomarker in the diagnostic process of these disorders^7^.

Bipolar Disorder is a chronic and recurrent disorder characterized by marked and persistent mood fluctuations^8^. These fluctuations, along with the associated symptoms, give rise to episodes of mania, hypomania, and depression^9^. Regarding the linguistic component, some studies have evidenced a generalized poverty of discursive content and deficits in referential abilities and social cognition^10,11^, with a higher frequency occurring during depressive states^12^. During manic phases, an increased number of wordplay and rhymes have been reported^13^. On the other hand, other studies have reported deficits in theory of mind abilities during both symptomatic and euthymic states, as well as general pragmatic deficits^14,15^. Noteworthy linguistic alterations include changes in speech rhythm, fluency, and content, as well as verbal memory impairments^16^. Different investigations^17,18^ have shown that these language deficits are due to cognitive impairment, primarily affecting attention, processing speed, verbal learning, memory, and executive functions.

Borderline Personality Disorder (BPD), on the other hand, is characterized by a pattern of instability in both affective and intrapersonal domains. This disorder is accompanied by cognitive impairments such as transient ideas, non-delusional referential thinking, and dissociative experiences^19^. Within the spectrum of BPD, there is a pattern shared by all individuals with this disorder, including turbulent relationships, emotional instability, and conflictual social interactions^20^. BPD is perhaps the psychopathological disorder that has received the least attention regarding language development. In this regard, some studies have demonstrated difficulties in understanding double meanings, metaphors, and irony frequently used as therapeutic intervention strategies^21^, as well as difficulties in inferring speaker's intentions^22,23^, deficits in constructing stable internal representations^24,25^, and a generalized pragmatic deficit^26^

Currently, while more researchers are focusing on delving into the relationship between language and psychopathology, the linguistic characteristics and differences in linguistic profiles among different pathologies remain an open debate^5,27^. In this regard, most studies so far seem to specifically focus on language deficits in individuals with schizophrenia^28–32^. However, the linguistic characteristics in individuals with other psychopathological disorders continue to be a controversial topic given the inherent complexity of human language and its interaction with thought^33–35^.

Given the limited research in this area, the objective of this study is to determine the linguistic difficulties in individuals with BPD and BD and, at the same time, compare the self-perception of individuals with BPD and BD with that of other informants, such as family members and therapists who work with them. As a working hypothesis, it was also proposed to know the relationship between communication skills and their prerequisites in the case of BD and BPD.

## Method

### Participants

The present study consisted of a total sample of 60 participants from Mental Health Centers in the southern region of the Community of Madrid, divided into two groups. The first group consisted of 31 individuals (26 females and 8 males) diagnosed with Borderline Personality Disorder (BPD), with a mean age of 30.1 years (SD = 5.61). The second group consisted of 29 individuals (14 females and 15 males) diagnosed with Bipolar Disorder (BD), with a mean age of 28.1 years (SD = 5.33).

Furthermore, considering that the symptomatology of both disorders could potentially bias the study, data regarding pragmatics were collected not only from the self-perception of the participants but also from their closest family members and referring professionals. Thus, 31 relatives of participants with BPD (18 mothers, 4 siblings, 9 partners) and 29 relatives of participants with BD (17 mothers and 12 partners) completed a questionnaire providing information on communicative competence from the perspective of the family members. Similarly, professionals who worked with the participants also provided information on pragmatic competence, with 31 questionnaires completed by social workers, psychologists, and psychiatrists for participants with BPD, and 29 questionnaires completed by psychiatrists, psychologists, and social workers for participants with BD.

### Instrument

Two assessment measures were used to gather data from the participants. The first measure was the Social Skills Scale (SSS)^36^. This questionnaire consists of 33 items that explore the individual's typical behavior in specific situations and assesses the extent to which social skills modulate these attitudes. It allows for the identification of the most problematic areas in terms of behaving assertively through a global index.

The Pragmatic Awareness Questionnaire (PAQ)^37^ was employed for this study. This questionnaire follows the integrative approach of the PerLa Corpus^38^ of clinical aphasia data and evaluation protocols such as the Revised Pragmatic Evaluation Protocol (PREP-R)^39^ and is inspired by the Pragmatic Protocol Manual by Prutting and Kirchner (1987)^40^.

The PAQ consists of blocks divided into 26 items distributed as ordinal variables on a 5-point Likert scale, where 1 = very poor, 2 = poor, 3 = fair, 4 = good, and 5 = very good. The wording of some items has been simplified, and examples have been added to facilitate comprehension. Each block of the questionnaire corresponds to a different area. Block I (items 1–2) evaluates intelligibility and paralanguage (intonation, volume, etc.). Block II (items 3–8) measures aspects of nonverbal communication, such as physical contact maintained during communicative acts or the distance established between interlocutors, including factors such as body posture, gaze, facial expression, and use of gestures. Block III (items 9–10) focuses on lexical competence and cohesion. Block IV (items 11–13) assesses semantic-pragmatic abilities related to the comprehension of irony and humor, as well as the interpretation of ambiguous utterances. Block V (items 14–16) pertains to morphosyntactic characteristics of discourse. Block VI (item 17) evaluates the ability to adapt to the interlocutor and communicative situation. Lastly, Block VII (items 18–27) focuses on the quantity and relevance of information in communicative exchanges, response time, control over turn-taking, the degree of acceptance and coherence of topics proposed by the subject, and conversation monitoring. The final item (26) is a general question about communication through an open-ended inquiry, where written perceptions and concerns regarding the level of pragmatic skills are collected.

Additionally, the Social Skills Scale (SSS)^36^ was used to assess the functionality of participants' pragmatic skills. This scale evaluates an individual's ability to modulate their attitude in everyday situations using socialization skills. It provides an index that identifies the specific areas (self-expression in social situations, defense of consumer rights, expression of anger or dissatisfaction, saying "no" and terminating interactions, making requests, and initiating positive interactions with the opposite sex) in which an individual may have difficulties behaving assertively.

### Procedure

To conduct this study, contact was made with different centers in the Community of Madrid that directly work with individuals diagnosed with BD and/or BPD. Initially, the centers were informed in person about the research project, and their interest in participating was solicited. Subsequently, potential participants were also informed. Once the participants received all the necessary information about voluntary participation and signed the informed consent form, they were scheduled for an individual session lasting approximately 20–30 min. In all cases, they were accompanied by a relevant professional who worked with them. In this session, the professional provided the participants with the questionnaires to be completed. Participants were allowed to ask questions about the items to ensure their understanding of what was being asked. To compare the obtained information, the questionnaires were completed, considering the different contexts in which the individual operates. Thus, the Pragmatic Competence Questionnaire was also completed by a family member (parent/mother/partner, etc.) and an active professional who intervened with individuals diagnosed with BD and/or BPD.

Therefore, 31 relatives of participants with BPD (18 mothers, 4 siblings, 9 partners) and 29 relatives of participants with BD (17 mothers and 12 partners) completed the questionnaire, providing information on communicative competence from the perspective of the family members. Likewise, information on pragmatic competence was obtained from professionals who completed 31 questionnaires for participants with BPD (6 social workers, 17 psychologists, and 8 psychiatrists) and 29 questionnaires for participants with BD (15 psychiatrists, 8 psychologists, and 6 social workers).

Finally, to control for certain biases that could influence the study, informants were asked to provide information about the subtype of BD and the medication being taken. Regarding the subtype of BD, all participants were diagnosed with Type II BD and were in a euthymic state. In terms of medication, participants in both groups were taking mood stabilizers, antidepressants, and antipsychotics. Antipsychotic medication was found in a lower proportion in the group of participants with BPD.

The study was conducted according to the guidelines of the Declaration of Helsinki. The Ethics Committee for Social Research at University of Castilla-La Mancha with reference CEIS-704512-L9M5 has issued a favourable opinion.

### Data analysis

For this research, statistical analysis was performed using SPSS 24.0. The normality of the sample was assessed using the Kolmogorov–Smirnov test, which indicated that the data were non-parametric. The independent samples t-test was used to determine if there were differences between the adolescent and adult groups. The Spearman's rho correlation coefficient was used to analyze the correlation between variables and informants.

## Results

The most relevant results regarding both groups are presented below. The data related to the Social Skills Scale can be observed in Table 1.

**Table 1:** Mean percentages on the Social Skills Scale.

Evaluated domain	BPD Group	BD Group
Self-expression in social situations	9.5 (6.0)	11.7 (8.8)
Defense of one's rights as a consumer	19.2 (14.9)	15.6 (13.1)
Expression of anger or dissatisfaction	39.8 (16.3)	33.8 (18.9)
Saying no and terminating interactions	22.6 (11.3)	22.4 (16.8)
Making requests	36.9 (23.1)	41.6 (30.0)
Initiating positive interactions with the opposite sex	33.6 (17.0)	32.0 (18.4)
Global score	14.8 (6.2)	14.8 (8.6)

Standard deviations in parentheses.

In the results described in Table 1, the mean percentages of achievement in the different evaluated areas can be observed. It is noteworthy that, although scores are generally very low in both groups in all areas (scoring below 40%), there are no significant differences between the group of individuals with BPD and the group of individuals with BD, except in the area of making requests where performance is higher in the BD group (p < 0.05).

Regarding the data obtained from the Pragmatic Awareness Questionnaire, it is important to note that, as previously indicated, this test was completed by three different informants: the individual with a diagnosis of BPD or BD (self-report), family members, and professionals working with the individual with BPD or BD. Table 2 presents the results of the self-report measures on their level of pragmatic awareness.

**Table 2:** Mean scores in the sections of the Pragmatic Awareness Questionnaire (self-report).

Evaluated items	BPD group	BD group
Intelligibility	2.26 (0.5)	2.07 (0.5)
Paralinguistic aspects	2.90 (0.9)	2.79 (1.1)
Distance in communication	3.45 (1.0)	3.62 (0.9)
Physical contact	2.97 (0.7)	2.79 (0–8)
Body posture	1.45 (0.6)	1.76 (0.8)
Use and performance of gestures	2.61 (0.9)	2.66 (1.0)
Facial expression	2.71 (0.8)	2.41 (0.8)
Eye gaze	1.42 (0.6)	1.31 (0.4)
Use of synonyms	2.97 (0.7)	3.14 (1.1)
Quantity of known and used words	2.42 (1.0)	2.69 (1.1)
Interpretation of ambiguous expressions and comments	2.74 (1.1)	2.69 (0.8)
Understanding and reaction to irony	2.77 (0.8)	3.00 (1.1)
Understanding and reaction to humor	3.52 (0.8)	3.31 (1.1)
Word construction	1.90 (0.8)	1.97 (0.8)
Appropriate grammatical structure	2.87 (0.9)	2.97 (1.2)
Ordered relation of ideas	2.61 (0.9)	2.62 (0.9)
Adaptation of communicative style to the context	2.90 (0.9)	2.66 (1.0)
Adjustment of topics in conversation	1.58 (0.6)	1.34 (0.5)
Topic changes in conversation	2.39 (0.8)	2.62 (1.1)
Maintenance and follow-up in conversation	2.81 (1.0)	2.69 (1.1)
Response time to a question	2.26(0.8)	2.59 (1.0)
Interruptions when other speakers are talking	3.77 (0.7)	3.69 (0.9)
Amount of information in the message	3.61 (0.9)	3.69 (1.1)
Understanding of other people in conversation	1.52 (0.7)	1.93 (0.9)
Empathy towards other people in conversation	1.61 (0.7)	1.90 (0.9)
Amount of information in communication	2.48 (0.9)	2.71 (1.0)

Standard deviations in parentheses.

In the case of self-report, it is observed that the items evaluated are very similar between both disorders. For example, the lowest scores in both the group of individuals with BPD and the group with BD are found in the areas of body posture, gaze, word construction, adjustment of conversation topics, and understanding of others and towards others in conversation. Similarly, regarding the areas in which strengths are shown, the trend is the same in both disorders. The areas of communication distance, understanding and reaction to humor, interruptions when other speakers are talking, and amount of information in the message stand out. However, there are two areas in which there are significant differences in favor of the BD group compared to BPD: use of synonyms (p < 0.01) and changes of topic in conversation (p < 0.05).

Regarding the questionnaire completed by family members, their perception also shows agreement in the area of the use of synonyms (p < 0.01) and changes of topic in conversation (p < 0.05) in favor of individuals with BD. Additionally, families perceive differences in the area of word construction (mean score BPD = 2.67; mean score BD = 2.76; p < 0.05). However, families perceive that in the area of adjustment of conversation topics, the performance is higher in the case of their relatives with BPD (mean score BPD = 2.55; mean score BD = 2.45; p < 0.05).

Finally, when analyzing the data from the perspective of the professionals involved in the intervention of both the BPD and BD groups, it is worth noting that professionals indicate that the existing differences between the two groups reside in the areas of intelligibility (mean score BPD = 2.71; mean score BD = 2.00; p < 0.01), where the performance is significantly higher in the BD group, and interpretation of ambiguous expressions and comments (mean score BPD = 2.61; mean score BD = 2.41; p < 0.05), where, conversely, the performance is significantly higher in the BPD group.

Regarding the intergroup comparison of the different evaluated areas based on the informants: individuals diagnosed with BPD/BD, families, and professionals, there are no significant differences among the three groups. Therefore, the perception of their pragmatic abilities is very similar in both groups.

## Discussion

The main objective of this study was to explore the self-perception of individuals with BPD and BD regarding their pragmatic competence and compare it with the perception of professionals working with them and their closest family members. We pursued this goal guided mainly by three reasons. First, we aimed to contribute new data to the debate regarding whether pragmatic language impairments in psychotic disorders have a primary origin or arise as a consequence of the well-established association between language and thought. Second, we wanted to assess the existence of impairments in social skills, hypothesizing that they could arise as a result of deficits in pragmatic-communicative abilities. Finally, given the scarcity of tools that assess pragmatic competence objectively and standardizedly, it was considered interesting to evaluate whether there were coincidences among the perception of professionals working with individuals diagnosed with BPD and BD, their own perception, and that of their families.

Although the results have shown that people with TB present difficulties in communication, the phase in which the person is should be taken into account, from depression to mania (BD-I) or hypomania (BD-II)^41,42^. For example, in the manic phase, patients usually present rushed and, in some cases, verbose language^43^. This can reach such a point of intensity and insistence that it is impossible for the recipient to influence the conversation and may even speak so fast that it is impossible to understand^44^. However, in the depressive phase it is common for the patient to answer with monosyllables or even remain silent for certain periods of time, which greatly hinders social communication^45^. However, although they may present a different profile, the linguistic alterations of this population have been evidenced once again^46^.

Regarding the first two questions, the results of our study have confirmed that both individuals with BPD and those with BD have limited social skills, in line with other studies^47–49^. Although most areas did not show significant differences in terms of social skills between individuals with BPD and BD, the results showed that individuals with BD made a greater number of requests. This could be explained as part of the characteristics of BPD: difficulties in making requests, assimilating refusals, and making prototypical rejections related to ambivalent or disorganized attachment and hypomentalization^50–54^. Thus, authors such as Bateman and Fonagy^55^ suggested the possibility that individuals with BPD might make a smaller number of demands due to difficulties in social cognition skills. In this sense, Bora^56^ and Bucci^57^ reported that hypomentalization in BPD manifests through marked withdrawal and poverty in reasoning skills. These difficulties seem to influence the development of symbolic function^58^ and, therefore, the ability to use metaphorical concepts that require transitioning from sub-symbolic to symbolic aspects of experience^57^.

About the third question, the results have shown a high level of agreement between self-perception of pragmatic difficulties, the perception of families, and the perception of professionals. This can be interpreted in line with studies that have addressed and confirmed the reliability of family members as informants of their relatives' abilities^59^. Furthermore, our results have revealed the existence of difficulties in factors associated with verbal language and factors associated with behavior within nonverbal communication, as well as in enunciative and interactive pragmatics in BPD^60,61^ and especially in BD^5,10,11,27^. In this regard, concerning BD, some studies found poorer performance in Theory of Mind (ToM) tasks and in elements of textual pragmatics in BD compared to individuals without psychopathic traits^5^, although a better performance compared to other psychotic disorders such as schizophrenia^62,63^. In contrast, authors such as^64^ conducted a systematic review in which they found that individuals with BD performed normally in tasks of naming, verbal competence, and word communication. Regarding BPD, no studies have been found that focus on studying the pragmatic skills of this population. However, some studies have reported communication difficulties as a result of relationship difficulties^65^. In this regard, Montigny-Malenfant et al.^66^ found that women diagnosed with BPD exhibited a greater number of dominant behaviors compared to their partners, which seemed to manifest through more overlapping and tangling, as well as a loss of pragmatic appropriateness and shared knowledge during conversation.

In conclusion, it is worth noting that although language development is not considered a research area of special interest within the spectrum of mental disorders, it is important to recognize that communication itself is a highly relevant factor for achieving greater autonomy and inclusion in society. Therefore, it should be taken into account when designing comprehensive and meaningful interventions for these populations.

## Data Availability

All data from this study are available upon request to the corresponding author.
